# Cs[Cl_3_F_10_]: A Propeller‐Shaped [Cl_3_F_10_]^−^ Anion in a Peculiar A^[5]^B^[5]^ Structure Type

**DOI:** 10.1002/anie.202007019

**Published:** 2020-08-17

**Authors:** Benjamin Scheibe, Antti J. Karttunen, Ulrich Müller, Florian Kraus

**Affiliations:** ^1^ Fachbereich Chemie Philipps-Universität Marburg Hans-Meerwein-Straße 4 35032 Marburg Germany; ^2^ Department of Chemistry and Materials Science Aalto University 00076 Aalto Finland

**Keywords:** density functional theory calculations, fluorides, halogens, interhalogen compound, X-ray diffraction

## Abstract

Reaction of CsF with ClF_3_ leads to Cs[Cl_3_F_10_]. It contains a molecular, propeller‐shaped [Cl_3_F_10_]^−^ anion with a central μ_3_‐F atom and three T‐shaped ClF_3_ molecules coordinated to it. This anion represents the first example of a heteropolyhalide anion of higher ClF_3_ content than [ClF_4_]^−^ and is the first Cl‐containing interhalogen species with a μ‐bridging F atom. The chemical bonds to the central μ_3_‐F atom are highly ionic and quite weak as the bond lengths within the coordinating *X*F_3_ units (X = Cl, and also calculated for Br, I) are almost unchanged in comparison to free *X*F_3_ molecules. Cs[Cl_3_F_10_] crystallizes in a very rarely observed A^[5]^B^[5]^ structure type, where cations and anions are each pseudohexagonally close packed, and reside, each with coordination number five, in the trigonal bipyramidal voids of the other.

## Graphical abstract

Reaction of CsF and ClF_3_ at low temperatures gives the compound Cs[Cl_3_F_10_] which crystallizes in a very rarely observed A^[5]^B^[5]^ structure type. A propeller‐shaped [Cl_3_F_10_]^−^ anion with a μ_3_‐bridging fluorine atom is present in the salt. Quantum‐chemical calculations show that the interaction of the ClF_3_ subunits with the μ_3_‐bridging fluorine atom are weak and the Cl−F bonds are essentially ionic.

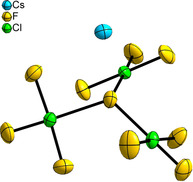

Fluorine containing interhalogen cations and anions derive from the neutral halogen fluorides. Those containing bridging μ‐F atoms are very scarce. Overviews of (homo and hetero) polyhalogen cations and anions have been given in recent review articles.^1^, ^2^, ^3^, ^4^, ^5^ So far, the only known species with μ‐bridging F atoms were reported for Br(III) and I(V). These are [Br_2_F_5_]^+^, [Br_3_F_8_]^+^, [Br_2_F_7_]^−^, [Br_3_F_10_]^−^, and [I_3_F_16_]^−^.^6^, ^7^, ^8^, ^9^, ^10^, ^11^, ^12^, ^13^ To the best of our knowledge, not a single Cl‐containing species, regardless of the oxidation state, with μ‐bridging F atoms is known. For the cationic side of Cl(III), only the [ClF_2_]^+^ cation is known and for the anionic side only tetrafluoridochlorates(III), *A*[ClF_4_], have been reported (*A*=K, Rb, Cs, NO, 1,1,3,3,5,5‐hexamethylpiperidinium).^14^, ^15^, ^16^, ^17^, ^18^ We present the synthesis and characterization of the first fluoridochlorate(III) anion with μ‐bridging F atoms. Figure 1 shows the Lewis structure of the propeller‐shaped [Cl_3_F_10_]^−^ anion and its molecular structure in the salt Cs[Cl_3_F_10_].

**Figure 1:**
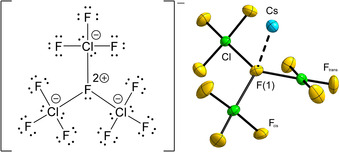
The Lewis structure of the [Cl_3_F_10_]^−^ anion and its structure in the salt Cs[Cl_3_F_10_]. The displacement ellipsoids are shown at the 70 % probability level at 100 K. Fluorine atoms drawn in yellow, chlorine atoms in green, and caesium atoms in sky blue. 41

Single crystals of the compound were obtained by reaction of CsF with excess liquid chlorine trifluoride at temperatures at or below 0 °C, see Equation (1).

(1) $$3\;\mathrm{ClF}{}_{3}+\mathrm{CsF}\overset{\mathrm{ClF}{}_{3}}{\underset{}{\to }}\mathrm{Cs}\left[\mathrm{Cl}{}_{3}F{}_{10}\right]$$

These crystals were directly isolated from cold ClF_3_ suspensions and handled at low temperatures under a nitrogen atmosphere. The crystals rapidly deteriorate upon warming to room temperature and/or moisture from air contact, releasing ClF_3_ and/or hydrolysis products. Further details on the synthesis are given in the Supporting Information. Single‐crystal X‐ray diffraction on Cs[Cl_3_F_10_] shows it to crystallize in the monoclinic space group *P*12_1_/*n*1 (*mP*56, 14, *e* ^14^). The lattice parameters are *a=*9.0949(7), *b=*15.3652(12), *c=*6.8841(5) Å, *β*=91.314(2)°, *V=*961.77(13) Å^3^, and *Z=*4 at *T=*100 K. Further crystallographic details are given in the Supporting Information, Tables S1–S5.

The compound contains the propeller‐shaped [Cl_3_F_10_]^−^ anion in *C*_1_ symmetry (Figure 1). However, the deviation of the molecule from *C*_3_ symmetry is small. The anion can be thought of as a central fluoride ion which is coordinated by the Cl atoms of three surrounding ClF_3_ molecules. The central μ_3_‐bridging F atom (F(1) in Figure 1) lies 0.522(2) Å above a virtual plane formed by the Cl atoms with Cl‐μ_3_‐F‐Cl angles from 113.53(9) to 116.12(8)°. Its structure is correctly predicted by VSEPR theory and there seems to be an additional interaction to the Cs^+^ counterion residing above the apex of the trigonal‐pyramidal anion (Figure 1).

In contrast, the quantum‐chemically calculated gas‐phase minimum structure is *D*_3_‐symmetric (TURBOMOLE, DFT‐PBE0/TZVP, see Supporting Information), that is the μ_3_‐F atom and the Cl atoms reside within a plane and the Cl‐μ_3_‐F‐Cl angles are 120°.^19^, ^20^, ^21^, ^22^ Even when the geometry optimization is started from *C*_3_ symmetry, where the μ_3_‐F atom is displaced from the virtual plane formed by the Cl atoms, the *D*_3_‐symmetric structure is reobtained.

In the crystal structure, the ClF_3_ units are essentially planar and are rotated around the μ_3_‐F−Cl bond in the same direction, which leads to the propeller shape of the anion. That is, the ClF_3_ units are tilted in comparison to the virtual plane formed by the Cl atoms by 33.34(7), 36.41(6), and 44.77(6)°. The anion is thus similarly shaped as the [Br_3_F_10_]^−^ anion, see below.^10^ The Cl atoms are surrounded by four F atoms each in a quadrilateral, almost planar manner. The μ_3_‐F−Cl distances are in the range of 2.243(2) to 2.265(2) Å. The F atoms *cis*‐bound relative to μ_3_‐F show F_cis_−Cl distances from 1.730(2) to 1.747(2) Å, while the *trans*‐bound F atoms show F_trans_−Cl distances of 1.600(2) to 1.615(2) Å. These are comparable with the F−Cl distances reported for the orthorhombic modification of ClF_3_ (1.716 and 1.621 Å) and thus support the description of the anion having a central fluoride anion coordinated by three ClF_3_ molecules.^23^ We will see below that the chemical bond between the μ_3_‐F atom and the three chlorine atoms is almost ionic. Also, a comparison with F−Cl distances in [ClF_4_]^−^ salts (1.794(4) to 1.8034(9) Å) shows significant differences which we attribute to the same reason.^17^, ^18^ In the structurally closely related [Br_3_F_10_]^−^ anions, the μ_3_‐F−Br distances range from 2.238(10) to 2.248(2) Å, the F_cis_−Br distances from 1.824(10) to 1.878(7) Å, and the F_trans_−Br distances from 1.745(2) to 1.767(7) Å.^10^ So, unexpectedly, the observed μ_3_‐F‐*X* distances are essentially similar. This is likely to be due to the direct interaction of the Cs^+^ cation with the μ_3_‐F atom in Cs[Cl_3_F_10_] leading to coordination number 3 + 1 for it, while such an interaction is absent in the crystal structure of Cs[Br_3_F_10_] and the coordination number of that μ_3_‐F atom is only three. The F_cis_‐*X* and F_trans_‐*X* atomic distances are circa 0.1 Å longer for *X*=Br compared to the Cl‐case, which agrees with the expectation.

As mentioned above, quantum‐chemical gas‐phase calculations for the homologous anions [*X*_3_F_10_]^−^ (*X*=Cl, Br, I) show them to be *D*_3_‐symmetric even when the geometry optimization is started from the pyramidal *C*_3_ symmetry around the μ_3_‐F atom as in the crystal structure. Except for the pyramidalization around the μ_3_‐F atom, the calculated structure of the *D*_3_‐symmetric [Cl_3_F_10_]^−^ anion nicely agrees with the atomic distances and angles reported here. The same is true for *X*=Br with the previously reported [Br_3_F_10_]^−^ anion in its Rb and Cs salts.^10^ To the best of our knowledge, a compound containing [I_3_F_10_]^−^ anions is not known yet. The calculated *X*‐F distances within the *X*F_3_ subunits are very similar to those in the solid‐state structures of the halogen trifluorides.^23^, ^24^, ^25^ This indicates a rather weak interaction of the *X*F_3_ units with the central μ_3_‐F atom. The calculated *X*‐μ_3_‐F distances of 2.22, 2.30 and 2.44 Å for *X*=Cl, Br, I, respectively, also support this.

To obtain a qualitative picture of the bonding situation in these anions we carried out an intrinsic atomic orbital (IAO) analysis to obtain partial atomic charges (Table S6) and generated the intrinsic bonding orbitals (IBOs), see Figure 2 and Figures S2–S4, which give information about the polarization and contribution of the atoms to the respective bond.^26^, ^27^ Further details are given in the Supporting Information. The obtained partial atomic charges from the IAO analysis show that the μ_3_‐F atom has the most negative partial charge in all three anions, followed by the F_cis_ atom of the *X*F_3_ units. The F_trans_ atoms are the least negatively partially charged F atoms. The partial charges of the atoms follow the expected trend in line with decreasing electronegativities *χ*_AR_(Cl) > *χ*_AR_(Br) > *χ*_AR_(I), that is, the iodine atoms are more positively partially charged than the Cl atoms and the F atoms are most negatively partially charged in [I_3_F_10_]^−^.^28^

**Figure 2:**
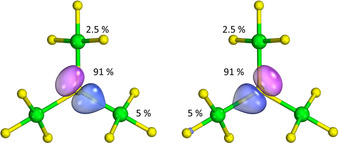
The two intrinsic bond orbitals (IBOs) showing the Cl‐μ_3_‐F‐Cl bonds in the [Cl_3_F_10_]^−^ anion.^27^ The percentages indicate the contribution of the Cl atoms and of the μ_3_‐F atom to the IBO. The larger the percentage, the more polarized the bond. For comparison: For a gas‐phase NaF molecule, in which the bond should be highly ionic, the contribution of the F atom is 96 %. In the H_2_ molecule, which has a purely covalent bond, the contribution of each atom is 50 %. If the summation of the percentages does not add up to 100 %, then other atoms contribute—less than 1 %—to the IBO. For further details, see the Supporting Information. F atoms: yellow, Cl atoms: green.

The two IBOs representing the Cl‐μ_3_‐F‐Cl bond in the [Cl_3_F_10_]^−^ anion are shown in Figure 2. Further IBOs showing the other F−Cl bonds, as well as the respective IBOs for [Br_3_F_10_]^−^ and [I_3_F_10_]^−^ are reported in the Supporting Information. The percentages next to the atoms show the contribution of each atom to the IBO. The higher the contribution of an atom to the IBO in %, the more ionic is the bond. The contribution of the μ_3_‐F atom to the respective IBO is above 90 % in all three anions, showing a highly polarized covalent, that is essentially ionic, bond (Figure 2 and Figures S2–S4). The heavier halogen atoms only have minor contributions to the IBO. Within the series, the contributions of the atoms follow the expected trend, which is due to the decrease of the electronegativities from Cl to I.

After the description of the molecular structure of the anion we come to the description of the crystal structure of Cs[Cl_3_F_10_].

Surprisingly, Cs[Cl_3_F_10_] can be regarded as a simple AB structure type. The Cs^+^ cations (“A”) are coordinated by five [Cl_3_F_10_]^−^ anions (“B”), that is C. N. (Cs^+^)=5, and that exclusively via the F_cis_ and μ_3_‐F atoms. The arrangement of the Cs cations corresponds to the hexagonal‐close packing with the layers arranged perpendicular to the *c* axis (Figure 3). Into the empty channels (parallel to the *c* axis), the terminally bound F_ter_ atoms protrude (see center of Figure 3). The μ_3_‐F atoms, which may be seen as the centers of gravity of the [Cl_3_F_10_]^−^ anions (“B”), are also arranged in a hexagonal‐close packed manner with the layers also perpendicular to the *c* axis. Their coordination number is also five. Thus, the two pseudohexagonal‐close packed lattices of A and B interpenetrate in such a way that each ion is surrounded by five of its counterions in a trigonal bipyramidal manner. That is, the packing of Cs[Cl_3_F_10_] can be described as a simple A^5^B^5^ structure type. Of course, the situation is somewhat more complicated because the [Cl_3_F_10_]^−^ ion is not spherical. But if we take idealized planar [Cl_3_F_10_]^−^ ions, the resulting space group is that of the hexagonal‐close packing, *P*6_3_/*mmc* (aristotype). Distortion of the planar [Cl_3_F_10_]^−^ ions to their real structure entails a symmetry reduction to the actual space group *P*12_1_/*n*1; that can be represented by the Bärnighausen tree^29^, ^30^, ^31^, ^32^, ^33^ shown in Figure S1. The crystal structure of the compound and its relation to the hexagonal aristotype is shown in Figure 3 and explained in detail in the Supporting Information.

**Figure 3:**
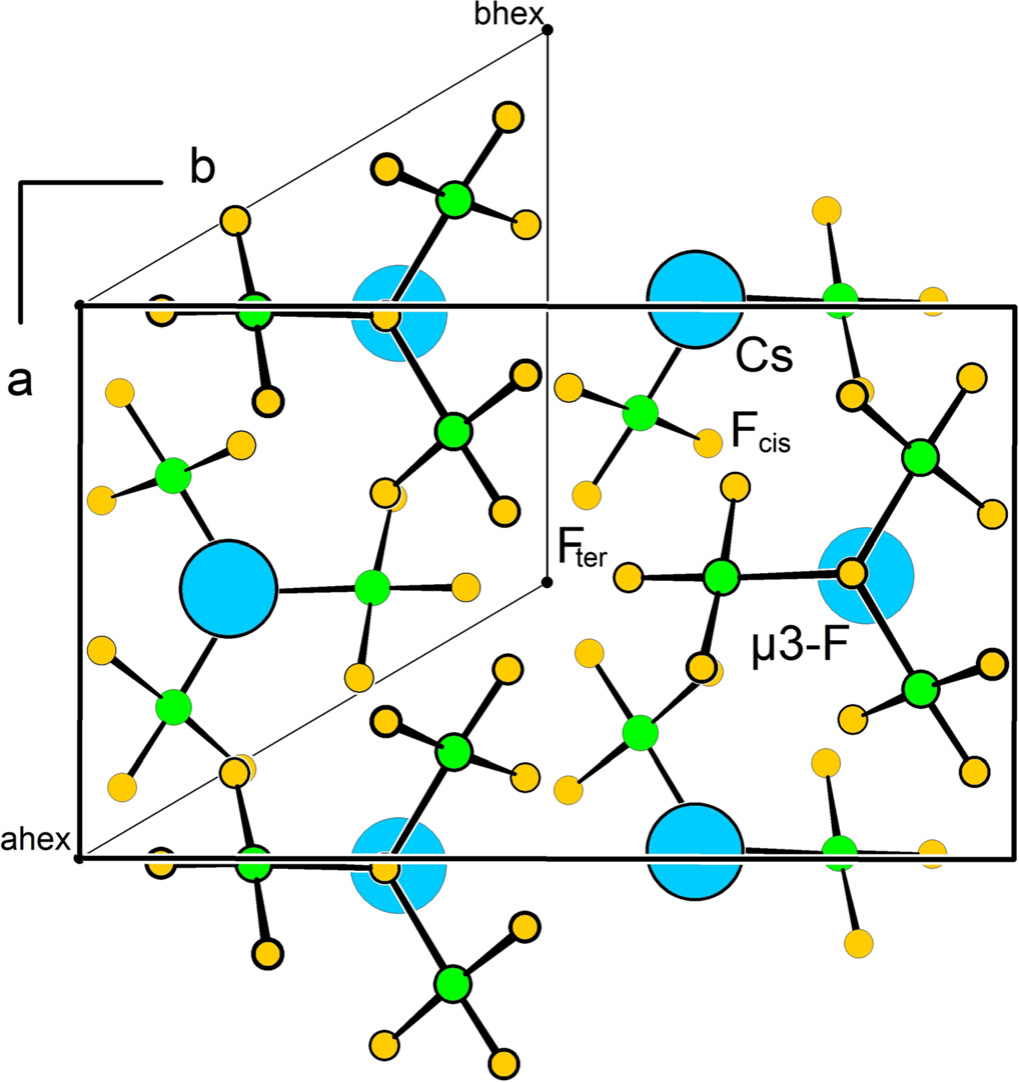
The crystal structure of Cs[Cl_3_F_10_] viewed along the *c* axis and its relation to the hexagonal aristotype. Cs atoms are shown in sky‐blue, Cl atoms in green, and F atoms in yellow. All atoms are drawn as spheres with arbitrary radii. Note the pseudohexagonal arrangement of the Cs^+^ cations and the [Cl_3_F_10_]^−^ anions. If the atoms in the pseudohexagonal cell would fulfil trigonal symmetry, the space group would be *P* $\bar{3}$ .^41^

A related structural motif of an A^5^B^5^ structure type is known to occur as a substructure in the Ni_2_In structure type, where one kind of Ni atoms reside in the octahedral voids and the other kind of Ni atoms in the trigonal bipyramidal voids of hexagonal‐close packed In atoms.^34^ The reported orthoselenate Li_4_ 5Se^5^O_5_ 5 is an ordered variant of the A^5^B^5^ aristotype.^35^, ^36^ Also, such a 5‐5 coordination has been quantum‐chemically predicted for a low temperature modification of sodium chloride Na^5^Cl^5^, and later also for magnesium oxide Mg^5^O^5^.^37^, ^38^ To the best of our knowledge, no binary compound with such a A^5^B^5^ crystal structure exists yet, and Cs[Cl_3_F_10_] appears to be one of the very rare examples of this type.

The recorded Raman spectrum of Cs[Cl_3_F_10_] along with a calculated one obtained from quantum‐chemical solid‐state calculations (CRYSTAL17, DFT‐PBE0/TZVP) is shown in Figure 4.^21^, ^22^, ^39^

**Figure 4:**
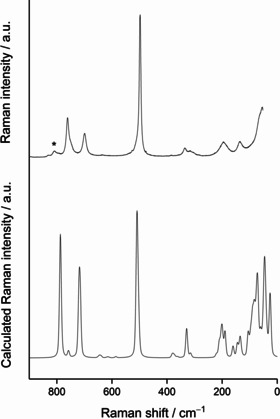
Top: The Raman spectrum of Cs[Cl_3_F_10_] recorded at circa −66 °C. The asterisk denotes a band caused by the perfluoropolyether used for the immersion of the sample. Bottom: Computational solid‐state Raman spectrum of Cs[Cl_3_F_10_]. The Raman intensities are given in arbitrary units, a.u. Further details are given in the Supporting Information.

A detailed comparison of the Raman spectra with the ones of Cs[ClF_4_] and liquid ClF_3_ as well as further details are given in the Supporting Information, Table S8 and Figure S5. The calculated Raman spectrum nicely agrees with the measured one, indicating that the main product obtained under the experimental conditions is Cs[Cl_3_F_10_]. The two main features of the Raman spectrum are the bands above 690 cm^−1^ and the band at approximately 500 cm^−1^. The first can be attributed to the Cl‐F_trans_ symmetric stretching mode and the latter to the Cl‐F_cis_ symmetric stretching mode.

In summary, we obtained the compound Cs[Cl_3_F_10_] which crystallizes in the very rarely observed A^5^B^5^ structure type. The cations and anions of Cs[Cl_3_F_10_] form interpenetrated hexagonal‐close packed layers. The coordination number of each cation and anion is five. The [Cl_3_F_10_]^−^ anion is propeller‐shaped with a symmetry close to *C*_3_ as the central μ_3_‐F atom is surrounded in trigonal‐pyramidal manner by the three Cl atoms, while quantum‐chemical gas‐phase calculations predict the anion to be *D*_3_‐symmetric with the μ_3_‐F atom in plane with the Cl atoms. The recorded Raman spectrum of Cs[Cl_3_F_10_] is in excellent agreement with the quantum‐chemically calculated one for the solid‐state. So far there is no experimental evidence for the existence of a [Cl_2_F_7_]^−^ anion that would be the analogue to the known [Br_2_F_7_]^−^ anion, or for anions with an even higher ClF_3_ content than [Cl_3_F_10_]^−^, such as a [Cl_4_F_13_]^−^ anion. Investigations to obtain compounds containing such anions are ongoing, as well as analogous ones that derive from ClF_5_.

## Conflict of interest

The authors declare no conflict of interest.

## Supporting information

As a service to our authors and readers, this journal provides supporting information supplied by the authors. Such materials are peer reviewed and may be re‐organized for online delivery, but are not copy‐edited or typeset. Technical support issues arising from supporting information (other than missing files) should be addressed to the authors.

Supplementary
