# Supplementary material for: Cs[Cl3F10]: A Propeller‐Shaped [Cl3F10]− Anion in a Peculiar A[5]B[5] Structure Type
Source: Angew Chem Int Ed Engl. 2020 Aug 17;59(41):18116–9. doi: 10.1002/anie.202007019 (PMC7589245; doi:10.1002/anie.202007019)
Supplement: Supplementary file 1 — Supplementary [file ANIE-59-18116-s001.pdf]

Angewandte Chemie
Eine Zeitschrift der Gesellschaft Deutscher Chemiker

Supporting Information

$Cs[Cl_3F_{10}]$: A Propeller-Shaped $[Cl_3F_{10}]^-$ Anion in a Peculiar $A^{[5]}B^{[5]}$ Structure Type

*Benjamin Scheibe, Antti J. Karttunen, Ulrich Müller, and Florian Kraus**

anie_202007019_sm_miscellaneous_information.pdf

Supporting Information
©Wiley-VCH 2019
69451 Weinheim, Germany

Table of Contents

Experimental Procedures 2
Selected crystallographic data of Cs[Cl_3F_{10}] 3
Crystallographic Considerations 7
Gas-phase quantum-chemical calculations of [X_3F_{10}]$^-$ (X = Cl, Br, I) anions 8
Solid-state quantum-chemical calculations of Cs[Cl_3F_{10}] 11
Band assignments for the calculated Raman spectrum of Cs[Cl_3F_{10}] and comparison of Raman spectra 12
References 14
Author Contributions........ 14

Experimental Procedures

General: All operations were performed on a Monel metal Schlenk line, which was passivated with undiluted fluorine and chlorine trifluoride at various pressures before use. Moisture-sensitive compounds were stored and handled in an Ar-filled glove box (MBraun). Reaction vessels were made out of fluoropolymer (perfluoroalkoxy alkanes, PFA) and passivated with fluorine before use. Preparations were carried out in an atmosphere of dry and purified argon (5.0, Praxair). Chlorine trifluoride was stored over NaF to remove traces of HF. **Caution!** Fluorine, chlorine trifluoride, and fluoridochlorates(III) must be handled using appropriate protective gear with ready access to proper emergency treatment procedures in the event of contact. The aforementioned are potent oxidative fluorinators that are only stable under the rigorously anhydrous conditions employed in the experimental procedures outlined in the Experimental Section. They react vigorously to explosively upon hydrolysis or contact with organic materials. The utmost precautions must be taken when disposing of these materials and their derivatives.

Synthesis of $Cs[Cl_3F_{10}]$: In a typical experiment a PFA reaction vessel was loaded with 46.8 mg CsF (0.308 mmol) inside a glove box and attached to a stainless-steel valve. An excess of ClF_3 (approximately 0.13 g, 1.4 mmol) was then condensed onto the solid at 77 K. The reaction mixture was slowly warmed to room-temperature, at which point the CsF completely dissolved. The resulting solution was then stored at −37 °C. Colorless crystals of $Cs[Cl_3F_{10}]$ grew from the solution after several hours at this temperature. Similar experiments were conducted at a temperature of 0 °C and the isolated crystals were determined to be $Cs[Cl_3F_{10}]$. The crystals were then transferred from the cold chlorine trifluoride suspensions into cold (<0 °C) Galden HT270 (perfluoropolyether, Solvay). Note that the crystals of $Cs[Cl_3F_{10}]$ were observed to be only stable at low temperatures (≤0 °C) and/or when suspended in chlorine trifluoride. The crystals of $Cs[Cl_3F_{10}]$ rapidly deteriorate upon warming to room temperature and air contact. When excess chlorine trifluoride was removed in vacuo, $Cs[ClF_4]$ was obtained, which was identified by Raman spectroscopy.

Single-crystal X-ray diffraction: Crystals of $Cs[Cl_3F_{10}]$ were selected in the absence of air under dried, cooled perfluoropolyether (Galden HT270, Solvay, stored over molecular sieves 3 Å) and mounted on a MiTeGen loop. Intensity data of suitable crystals were recorded with a D8 Quest diffractometer (Bruker). The diffractometer was operated with monochromatized Mo-K_α radiation (0.71073 Å, multi layered optics) and equipped with a PHOTON 100 CMOS detector. Evaluation, integration, and reduction of the diffraction data was carried out with the APEX3 software suite.[1] Split reflections were observed for multiple specimens, which indicate non-merohedral twinning. The measured crystal was a non-merohedral twin with three components. The cell constants and twin laws were determined with the program CELL_NOW.[2] The diffraction data were corrected for absorption utilizing the multi-scan method of TWINABS within the APEX3 software suite. For the structure solution only the non-overlapping reflections of the major twin component were used. The structure was solved with dual-space methods (SHELXT).[3] The data were initially refined with the HKLF5 format option in SHELXL with all reflections (overlapping reflections and non-overlapping reflections of the three twin components).[4] The data were then processed with the HKLF5Tools program: the non-overlapping reflections of the weaker diffracting twin components were removed, the non-overlapping reflections of the major twin component were merged in point group 2/*m* and the overlapping reflections were merged in point group 1. The final refinement was carried out against the detwinned dataset (created by SHELXL with the LIST 8 option as a FCF file and converted to a HKL file with HKLF5Tools).[5] The highest residual electron density after the final refinement was 0.78 Å distant from atom Cs(1). Representations of the crystal structure were created with the Diamond software.[6] CCDC 1992456 contains the supplementary crystallographic data for this paper. These data are provided free of charge by The Cambridge Crystallographic Data Centre.

Raman spectroscopy: The Raman spectra were measured with a Monovista CRS+ confocal Raman microscope (Spectroscopy & Imaging GmbH) using a 532 nm solid-state laser and either a 300 grooves/mm (low-resolution mode, FWHM: <4.62 cm^{-1}) or an 1800 grooves/mm (high-resolution mode, FWHM: <0.368 cm^{-1}) grating. Sample preparation of $Cs[Cl_3F_{10}]$: A crop of colorless crystals was quickly transferred from a cold chlorine trifluoride suspension (ca. −37 °C) into Galden HT270 (perfluoropolyether, Solvay) on a microscope slide with a recess. The microscope slide was located slightly above a small Dewar vessel, which was filled with liquid nitrogen. Raman spectra of the crystals were recorded as soon as the perfluoropolyether was a viscous liquid (approximately −66 °C, which is the pour point of Galden HT270).

Selected crystallographic data of $Cs[Cl_3F_{10}]$

Determination of the cell constants and twin law using CELL_NOW[2]

CELL_NOW output:

The following cells would appear to be plausible, but should be checked using
XPREP because they are not necessarily the conventional cells.

FOM, % within 0.2, a..gamma, volume and lattice type for potential unit-cells:

1	1.000	78.1	6.887	15.380	9.089	90.01	91.21	89.93	962.5	P
2	0.651	78.1	6.887	15.380	11.288	90.02	126.41	89.93	962.3	P
3	0.494	79.7	6.887	9.089	16.848	89.50	65.93	88.79	962.7	P
4	0.420	79.3	6.887	9.089	17.868	59.43	89.44	88.79	962.8	P
5	0.416	80.6	6.887	9.089	19.215	61.27	68.44	88.79	962.9	P
6	0.393	77.5	6.887	9.089	16.856	89.48	65.82	88.79	962.3	P
7	0.330	78.2	6.887	9.089	19.082	117.94	110.60	91.21	962.6	P
8	0.327	77.9	11.523	15.380	16.343	90.03	94.16	89.97	2888.9	P
9	0.324	79.8	6.887	11.288	16.848	75.99	65.93	53.59	962.5	P
10	0.315	76.5	6.887	9.089	17.865	59.41	89.34	88.79	962.4	P
11	0.305	77.2	11.288	15.380	16.669	90.04	93.34	89.98	2888.9	P
12	0.301	77.4	6.887	15.380	22.575	90.04	126.41	89.93	1924.7	P

Cell for domain 1: 6.887 15.380 9.089 90.01 91.21 89.93

Figure of merit: 0.845 %(0.1): 74.7 %(0.2): 78.1 %(0.3): 82.7

Orientation matrix:
0.09446988 -0.02510910 0.07350846
0.02914441 -0.04837650 -0.06969371
0.10637643 0.03545101 -0.04302402

Percentages of reflections in this domain not consistent with lattice types:
A: 36.2, B: 51.0, C: 49.1, I: 48.8, F: 68.1, O: 66.9 and R: 67.2%

Percentages of reflections in this domain that do not have:
h=2n: 49.9, k=2n: 51.4, l=2n: 49.6, h=3n: 67.3, k=3n: 68.0, l=3n: 66.8%

19783 reflections within tolerance assigned to domain 1,
19783 of them exclusively; 4897 reflections not yet assigned to a domain
----

Cell for domain 2: 6.887 15.380 9.089 90.01 91.21 89.93

Figure of merit: 0.470 %(0.1): 46.7 %(0.2): 52.7 %(0.3): 63.4

Orientation matrix:
0.09041524 -0.02595982 -0.07266141
0.02975903 0.05938596 -0.03815977
0.10967856 0.00518869 0.07332070

Rotated from first domain by 118.8 degrees about
reciprocal axis 1.000 0.002 -0.028 and real axis 1.000 0.000 0.000

Twin law to convert hkl from first to this domain (SHELXL TWIN matrix):
1.000 0.000 0.000
-0.038 -0.482 -1.483
-0.042 0.518 -0.482

12849 reflections within tolerance assigned to domain 2,
2828 of them exclusively; 2069 reflections not yet assigned to a domain
----

Cell for domain 3: 6.887 15.380 9.089 90.01 91.21 89.93

Figure of merit: 0.704 %(0.1): 69.7 %(0.2): 71.2 %(0.3): 76.5

Orientation matrix:
-0.09413844 0.04955499 -0.00076555
-0.02969191 -0.01075762 -0.10662626
-0.10651876 -0.04069526 0.02724020

Rotated from first domain by 179.9 degrees about
reciprocal axis -0.014 0.961 1.000 and real axis 0.003 0.335 1.000

Twin law to convert hkl from first to this domain (SHELXL TWIN matrix):
-1.000 -0.007 -0.020
0.007 -0.513 1.453
0.004 0.507 0.513

10441 reflections within tolerance assigned to domain 3,
1505 of them exclusively; 564 reflections not yet assigned to a domain

Compared to the output of CELL_NOW the unit cell was transformed according to **c**, –**b**, **a** and correspondingly *l* –*k*, *h* . This way, the monoclinic *c* axis coincides with the pseudohexagonal *c* axis and the monoclinic unit cell corresponds to the orthohexagonal cell. The axes of the twin domains mentioned in the output of CELL_NOW have to be relabeled accordingly.

Table S1. Selected crystallographic data and details of the structure determination of $Cs[Cl_3F_{10}]$.

Formula	$CsCl_3F_{10}$
Molar mass / g·mol^{-1}	429.26
Space group (No.)	$P12_1/n1$ (14)
a / Å	9.0949(7)
b / Å	15.3652(12)
c / Å	6.8841(5)
β / °	91.314(2)
V / Å^3	961.77(13)
Z	4
Pearson symbol	$mP56$
$\rho_{calc.}$ / g·cm^{-3}	2.97
μ / mm^{-1}	4.795
Color	colorless
Crystal morphology	block
Crystal size / mm^3	0.060 · 0.082 · 0.170
T / K	100
λ / Å	0.71073 (Mo-K$_\alpha$)
No. of reflections	72465
θ range / °	2.60-30.50
Range of Miller indices	$-12 \leq h \leq 12$
	$-21 \leq k \leq 21$
	$-9 \leq l \leq 9$
Absorption correction	multi-scan
T_{max}, T_{min}	0.75, 0.51
R_{int}, R_σ	0.050, 0.0471
Completeness of the data set	0.996
No. of unique reflections	2915
No. of parameters	127
No. of restraints	0
No. of constraints	0
S (all data)	1.09
$R(F)$ ($I \geq 2\sigma(I)$, all data)	0.0353, 0.0459
$wR(F^2)$ ($I \geq 2\sigma(I)$, all data)	0.0819, 0.0863
Extinction coefficient	not refined
$\Delta\rho_{max}$, $\Delta\rho_{min}$ / e·Å^{-3}	2.29, −1.26

Table S2. Atomic coordinates and equivalent isotropic displacement parameters U_{iso} of $Cs[Cl_3F_{10}]$ at 100 K.

Atom	Position	*x*	*y*	*z*	U_{iso} / Å^2
Cs(1)	4*e*	0.01291(2)	0.34115(2)	0.26558(3)	0.02195(9)
Cl(1)	4*e*	0.00999(7)	0.18673(5)	0.79210(11)	0.01919(16)
Cl(2)	4*e*	0.22709(8)	0.39970(5)	0.77877(11)	0.01866(16)
Cl(3)	4*e*	−0.19246(8)	0.40043(5)	0.75364(10)	0.01628(15)
F(1)	4*e*	0.0182(2)	0.32657(13)	0.6995(3)	0.0238(4)
F(2)	4*e*	−0.1537(2)	0.16571(15)	0.6662(4)	0.0417(6)
F(3)	4*e*	0.0091(2)	0.08724(12)	0.8674(3)	0.0352(5)
F(4)	4*e*	0.1731(2)	0.20129(14)	0.9274(3)	0.0338(5)
F(5)	4*e*	0.3386(3)	0.32653(14)	0.6604(4)	0.0402(6)
F(6)	4*e*	0.3719(2)	0.45434(14)	0.8376(3)	0.0364(5)
F(7)	4*e*	0.1245(2)	0.47666(14)	0.9043(3)	0.0283(5)
F(8)	4*e*	−0.1472(2)	0.47675(14)	0.5765(3)	0.0298(5)
F(9)	4*e*	−0.3422(2)	0.45278(14)	0.7841(3)	0.0327(5)
F(10)	4*e*	−0.2477(2)	0.32775(13)	0.9320(3)	0.0329(5)

Table S3. Anisotropic displacement parameters of $Cs[Cl_3F_{10}]$ at 100 K.

Atom	U_{11} / Å^2	U_{22} / Å^2	U_{33} / Å^2	U_{23} / Å^2	U_{13} / Å^2	U_{12} / Å^2
Cs(1)	0.02457(12)	0.02174(13)	0.01951(12)	0.00040(7)	−0.00004(8)	−0.00298(7)
Cl(1)	0.0185(3)	0.0186(4)	0.0202(3)	−0.0026(3)	−0.0047(3)	0.0022(2)
Cl(2)	0.0193(3)	0.0165(4)	0.0205(4)	0.0012(3)	0.0067(3)	0.0026(3)
Cl(3)	0.0149(3)	0.0159(3)	0.0179(3)	−0.0002(2)	−0.0024(2)	−0.0002(2)
F(1)	0.0217(9)	0.0226(10)	0.0269(10)	0.0031(8)	−0.0015(7)	0.0053(7)
F(2)	0.0291(12)	0.0431(14)	0.0521(16)	−0.0145(11)	−0.0209(11)	−0.0006(9)
F(3)	0.0389(12)	0.0168(10)	0.0497(14)	0.0020(10)	−0.0023(10)	0.0002(9)
F(4)	0.0285(11)	0.0343(13)	0.0378(12)	0.0115(9)	−0.0177(9)	−0.0046(9)
F(5)	0.0434(14)	0.0261(11)	0.0521(15)	−0.0031(10)	0.0274(12)	0.0092(10)
F(6)	0.0194(9)	0.0311(12)	0.0591(16)	−0.0026(10)	0.0092(10)	−0.0078(8)
F(7)	0.0239(10)	0.0283(11)	0.0328(11)	−0.0117(9)	0.0030(9)	0.0053(8)
F(8)	0.0335(11)	0.0240(11)	0.0317(11)	0.0090(9)	−0.0014(9)	0.0005(9)
F(9)	0.0192(9)	0.0345(12)	0.0445(13)	0.0025(10)	0.0016(9)	0.0098(8)
F(10)	0.0344(12)	0.0338(12)	0.0306(12)	0.0094(9)	0.0071(9)	−0.0024(9)

Table S4. Selected experimental and computational (DFT-PBE0/TZVP, solid-state and DFT-PBE0/TZVP, gas-phase, D_3 symmetry) atom distances for the $[Cl_3F_{10}]^-$ anion.

	d (experimental) / Å	*d* (DFT-PBE0, solid-state) / Å	*d* (DFT-PBE0, gas-phase) / Å
Cl(1)−F(1)	2.243(2)		
Cl(2)−F(1)	2.263(2)	2.24-2.27	2.22
Cl(3)−F(1)	2.265(2)		
Cl(1)−F(2)	1.736(2)		
Cl(1)−F(4)	1.747(2)		
Cl(2)−F(5)	1.730(2)	1.74-1.76	1.73
Cl(2)−F(7)	1.747(2)		
Cl(3)−F(8)	1.747(2)		
Cl(3)−F(10)	1.743(2)		
Cl(1)−F(3)	1.615(2)		
Cl(2)−F(6)	1.606(2)	1.60-1.62	1.64
Cl(3)−F(9)	1.600(2)		

Table S5. Selected experimental and computational (DFT-PBE0/TZVP, solid-state and DFT-PBE0/def2-TZVP, gas-phase D_3 symmetry) angles for the $[Cl_3F_{10}]^-$ anion.

	∡ (experimental) / °	∡ (DFT-PBE0, solid-state) / °	∡ (DFT-PBE0, gas-phase) / °
Cl(1)−F(1)−Cl(2)	116.12(8)		
Cl(2)−F(1)−Cl(3)	114.83(9)	109.8-115.5	120
Cl(3)−F(1)−Cl(1)	113.52(9)		
F(1)−Cl(1)−F(3)	177.29(10)		
F(1)−Cl(2)−F(6)	178.03(10)	177.2-178.1	180
F(1)−Cl(3)−F(9)	178.03(10)		
F(2)−Cl(1)−F(4)	176.13(12)		
F(5)−Cl(2)−F(7)	176.42(12)	176.1-176.7	174.5
F(8)−Cl(3)−F(10)	176.47(11)		
F(2)−Cl(1)−F(3)	88.50(11)		
F(3)−Cl(1)−F(4)	87.73(11)		
F(5)−Cl(2)−F(6)	88.41(12)	87.8-88.6	87.3
F(6)−Cl(2)−F(7)	88.05(11)		
F(8)−Cl(3)−F(9)	88.19(11)		
F(9)−Cl(3)−F(10)	88.28(11)		

Crystallographic Considerations

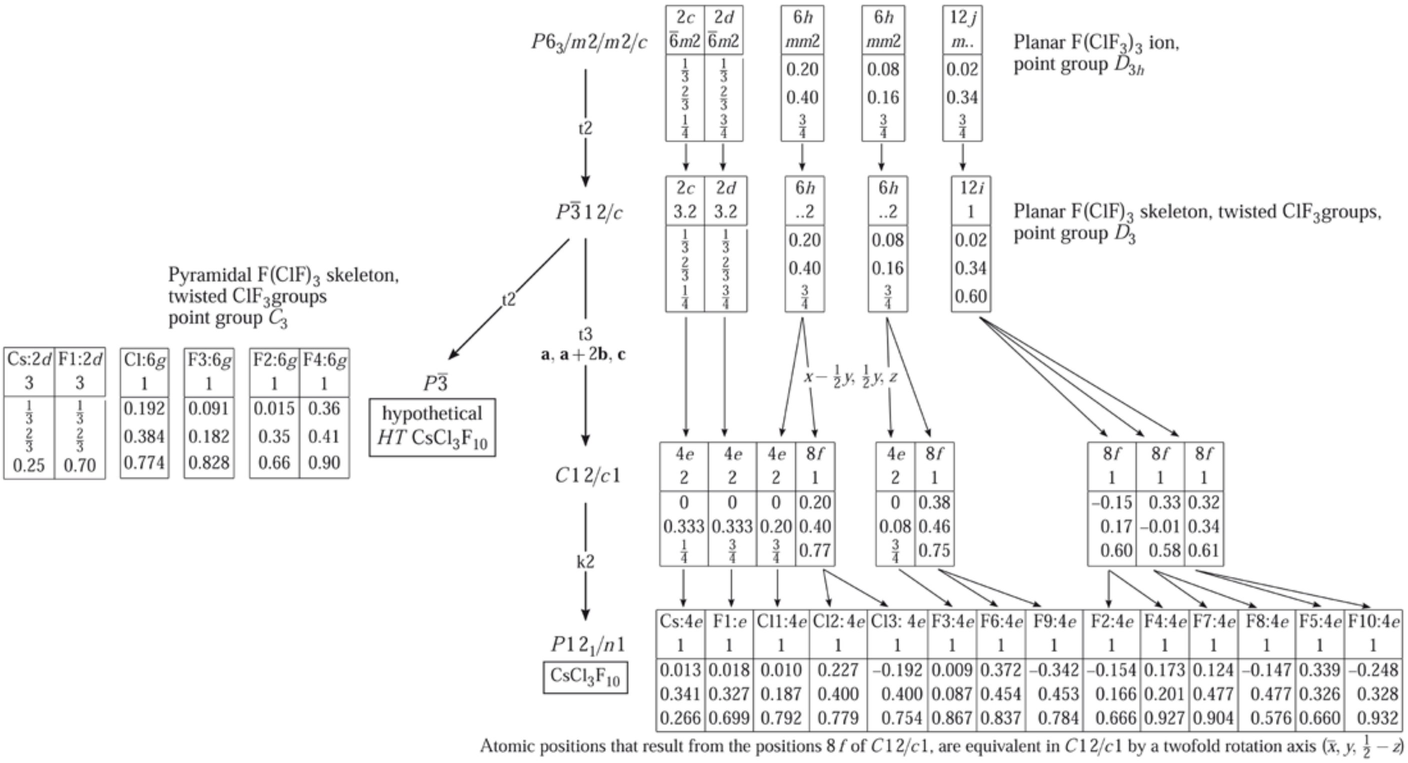

Figure S1. Bärnighausen tree showing the group–subgroup relations from a hexagonal aristotype with Cs atoms arranged as in the Mg type and with planar $[Cl_3F_{10}]^-$ in its trigonal pyramidal voids to the real structure of. $Cs[Cl_3F_{10}]$. The branch to the space group $P\overline{3}$ shows the relation to a hypothetical high-temperature form of $Cs[Cl_3F_{10}]$.

The actual crystal structure corresponds in good approximation to a hypothetical structure in the space group $P\overline{3}$, which is another subgroup of $P6_3/mmc$ (Figure S1). It seems probable that this could be a trigonal high-temperature modification of $Cs[Cl_3F_{10}]$ that, however, possibly cannot be obtained because of its instability at temperatures higher than 0 °C. This is in accordance with the observed twinning. As mentioned above (Selected crystallographic data), there exists a twin domain 3 rotated by 180° about *a* and another twin domain 2 rotated by 120° about *c*. That is exactly what is to be expected from the Bärnighausen tree, where we have a *translationengleiche* subgroup of index 2 (marked t2) giving rise to twins, and another one of index 3 (t3) giving rise to triplet twinning. Since the space group $P\overline{3}$ is not a direct supergroup of $P12_1/n1$, a phase transformation from the real to the hypothetical high-temperature form could only be a first-order transformation.

Gas-phase quantum-chemical calculations of $[X_3F_{10}]^-$ (X = Cl, Br, I) anions

Gas-phase quantum-chemical calculations for the anions $[X_3F_{10}]^-$ (X = Cl, Br, I) were carried out with the TURBOMOLE program package (version 7.3.) using the PBE0 hybrid density functional method and Karlsruhe triple-zeta-valence + polarization basis sets (def2-TZVP for Cl and Br, dhf-TZVP for I).[7–9] The resolution-of-the-identity technique was used to speed up the calculations.[10,11] All calculations were done in the gas phase without any solvent models. A full geometry optimization was carried out in the D_3 point group. All structures were confirmed to be true local minima by harmonic frequency calculations. The bonding in the studied species was investigated with the help of Intrinsic Atomic Orbitals (IAOs) and Intrinsic Bond Orbitals (IBOs) using the program package IboView.[12,13] The plotted IBO isosurfaces enclose 80% of the total electron density of the IBO.

XYZ coordinates of the geometry-optimized $[Cl_3F_{10}]^-$ anion

Cl 1.1077871 −1.9187436 0.0000000
Cl 1.1077871 1.9187436 0.0000000
F 0.0000000 0.0000000 0.0000000
F 0.1358137 2.5755586 −1.2780215
F 1.9275393 −3.3385960 0.0000000
F 2.1625923 1.4053974 1.2780215
F 2.1625923 −1.4053974 −1.2780215
F 0.1358137 −2.5755586 1.2780215
F 1.9275393 3.3385960 0.0000000
Cl −2.2155742 0.0000000 0.0000000
F −3.8550786 0.0000000 0.0000000
F −2.2984060 1.1701612 1.2780215
F −2.2984060 −1.1701612 −1.2780215

XYZ coordinates of the geometry-optimized $[Br_3F_{10}]^-$ anion

Br 1.1496286 −1.9912152 0.0000000
Br 1.1496286 1.9912152 0.0000000
F −0.0000000 0.0000000 0.0000000
F 0.1667520 2.6976675 −1.3923404
F 2.0305703 −3.5170509 0.0000000
F 2.2528726 1.4932452 1.3923404
F 2.2528726 −1.4932452 −1.3923404
F 0.1667520 −2.6976675 1.3923404
F 2.0305703 3.5170509 0.0000000
Br −2.2992573 0.0000000 0.0000000
F −4.0611406 0.0000000 0.0000000
F −2.4196246 1.2044223 1.3923404
F −2.4196246 −1.2044223 −1.3923404

XYZ coordinates of the geometry-optimized $[I_3F_{10}]^-$ anion

I 1.2209583 −2.1147618 0.0000000
I 1.2209583 2.1147618 0.0000000
F 0.0000000 0.0000000 0.0000000
F 0.3666149 2.8334558 −1.6241675
F 2.1708407 −3.7600063 0.0000000
F 2.2705373 1.7342257 1.6241675
F 2.2705373 −1.7342257 −1.6241675
F 0.3666149 −2.8334558 1.6241675
F 2.1708407 3.7600063 0.0000000
I −2.4419166 0.0000000 0.0000000
F −4.3416813 0.0000000 0.0000000
F −2.6371521 1.0992301 1.6241675
F −2.6371521 −1.0992301 −1.6241675

Table S6. Partial atomic charges for the $[X_3F_{10}]^-$ (X = Cl, Br, I) anions from Intrinsic Atomic Orbital (IAO) analysis (e^- units).

Atom	$[Cl_3F_{10}]^-$	$[Br_3F_{10}]^-$	$[I_3F_{10}]^-$
X	1.29	1.44	1.68
μ_3-F	−0.60	−0.62	−0.67
F_{trans}	−0.39	−0.45	−0.55
F_{cis}	−0.52	−0.56	−0.62

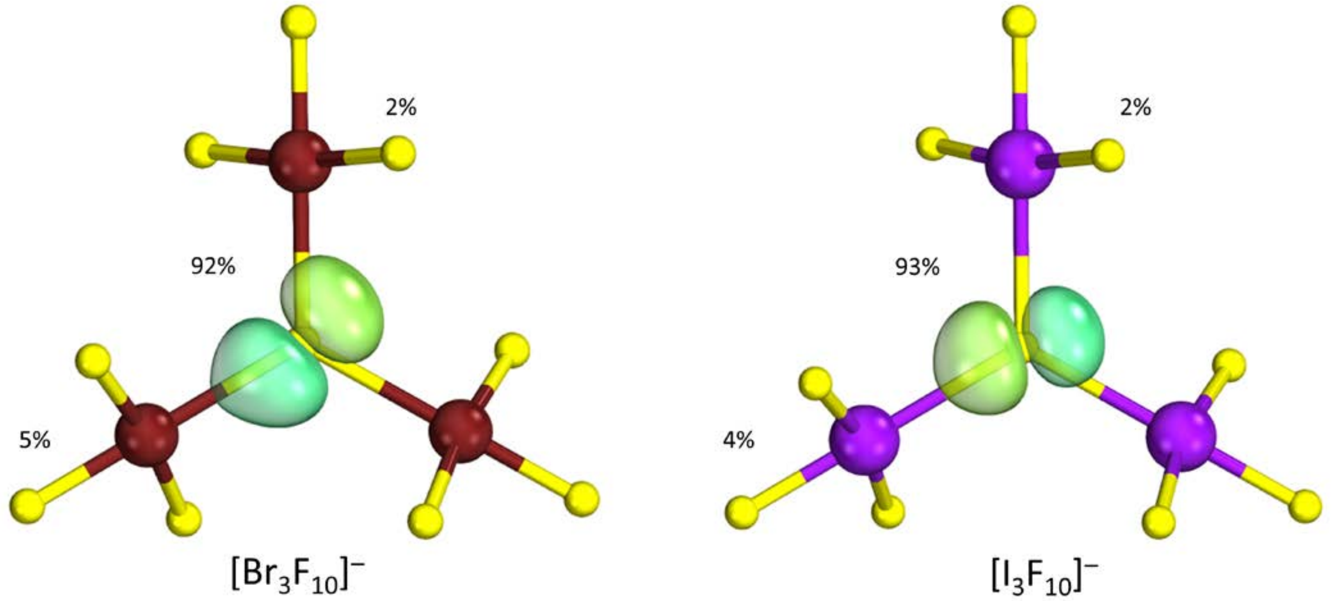

Figure S2. One of the two intrinsic bond orbitals (IBOs) showing the μ_3-F−*X* bonds in the $[Br_3F_{10}]^-$ and $[I_3F_{10}]^-$ anions (*X* = Br, I).[22] The percentages indicate the contribution of the halogen atoms and the μ_3-F atom to the IBO. The larger the percentage, the more polarized the bond. For comparison: For a gas-phase NaF molecule, in which the bond should be highly ionic, the contribution of the F atom is 96 %. In the H_2 molecule, which has a purely covalent bond, the contribution of each atom is 50 %. If the summation of the percentages does not add up to 100 %, then other atoms contribute – less than 1 % – to the IBO. F atoms: yellow, Br atoms: reddish brown, I atoms: purple.

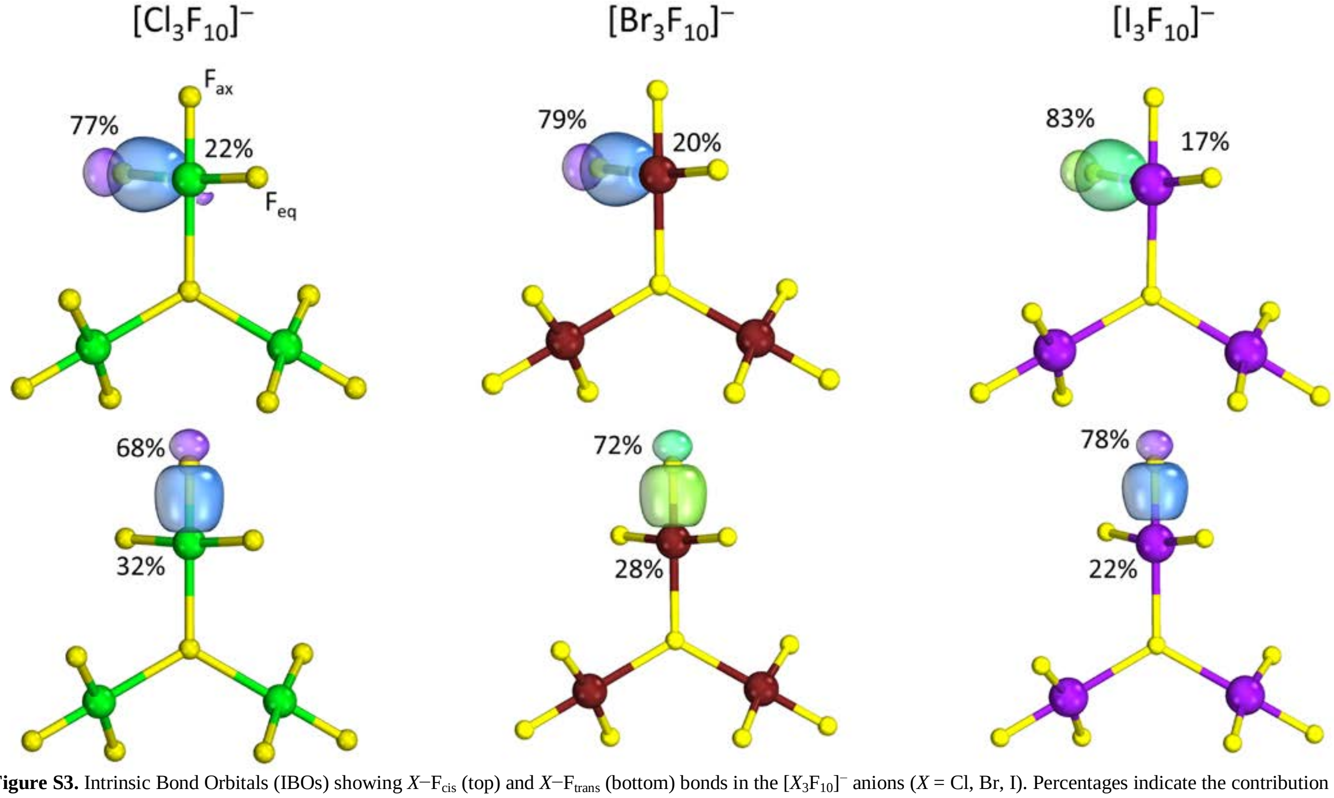

Figure S3. Intrinsic Bond Orbitals (IBOs) showing *X*−F_{cis} (top) and *X*−F_{trans} (bottom) bonds in the $[X_3F_{10}]^-$ anions (*X* = Cl, Br, I). Percentages indicate the contribution of each atom to the IBO. The larger the percentage, the more polarized the bond. For comparison: For a gas-phase NaF molecule, in which the bond should be highly ionic, the contribution of the F atom is 96 %. In the H_2 molecule, which has a purely covalent bond, the contribution of each atom is 50 %. If the summation of the percentages does not add up to 100 %, then other atoms contribute – less than 1 % – to the IBO. F atoms: yellow, Cl atoms: green, Br atoms: reddish brown, I atoms: purple.

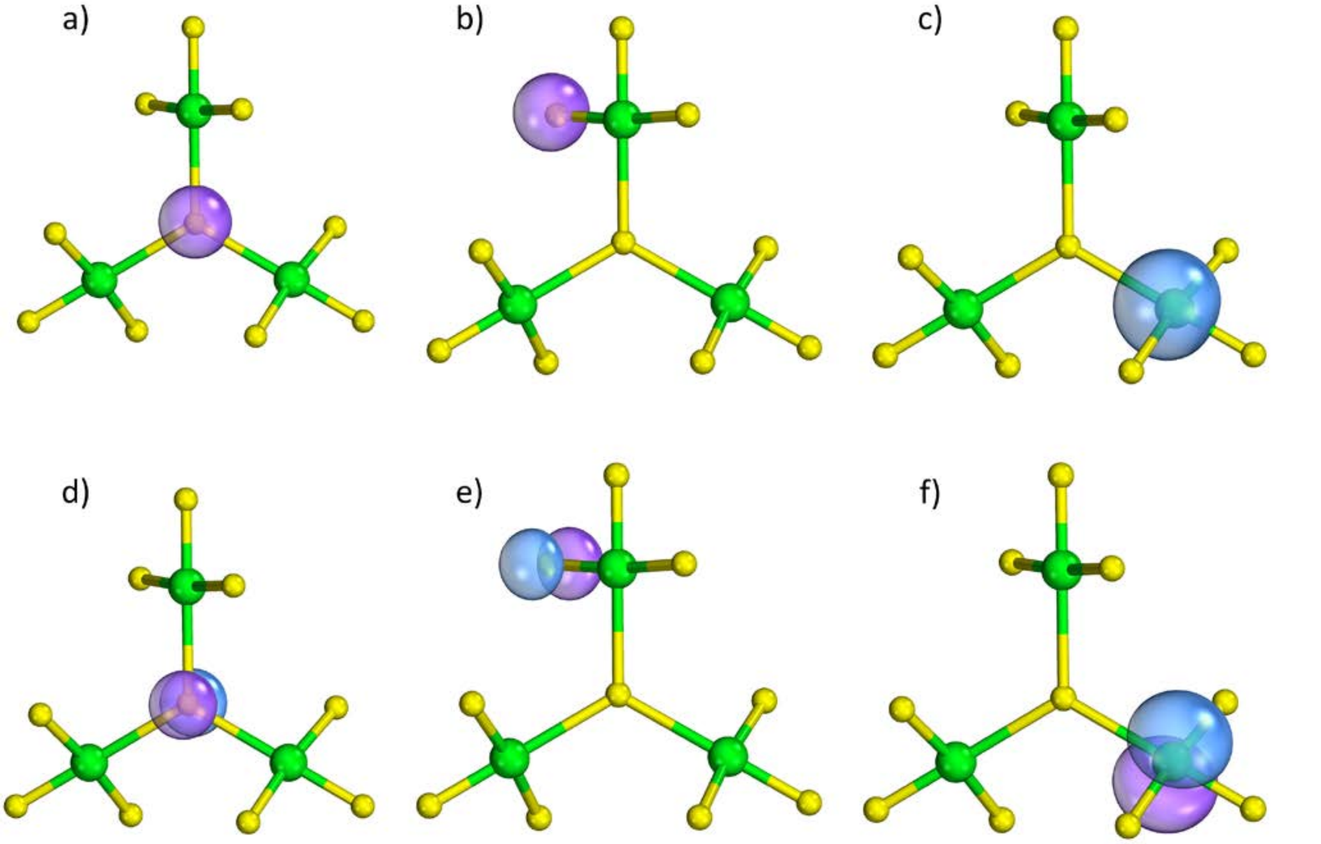

Figure S4. Examples of non-bonding Intrinsic Bond Orbitals (IBOs) in the $[Cl_3F_{10}]^-$ anion. s-orbital-like IBOs are shown in a)-c). p-orbital-like IBOs are shown in d)-f). F atoms in yellow, Cl atoms in green.

Solid-state quantum-chemical calculations of Cs[Cl_3F_{10}]

Periodic quantum-chemical calculations were carried out for Cs[Cl_3F_{10}] with the PBE0 hybrid density functional theory method (DFT-PBE0).[14,15] A triple-zeta-valence + polarization (TZVP) level basis set was applied for F and Cl and a split-valence + polarization (SVP) level basis set was applied for Cs. The basis sets were derived from the Karlsruhe def2 basis sets and taken from previous studies.[9,16–18] All calculations were carried out with the CRYSTAL17 program package.[19] The reciprocal space was sampled with a 4×2×3 Monkhorst-Pack-type *k*-point grid. For the evaluation of the Coulomb and exchange integrals (TOLINTEG), tight tolerance factors of 8, 8, 8, 8, 16 were used for all calculations. Both the atomic positions and lattice parameters were fully optimized within the constraints imposed by the space group symmetry. Default DFT integration grids and optimization convergence thresholds were applied in all calculations. The harmonic vibrational frequencies and Raman intensities were obtained through usage of the computational scheme implemented in CRYSTAL17.[20–23] The Raman intensities were calculated for a polycrystalline powder sample (total isotropic intensity in arbitrary units). The Raman spectra were obtained by using a pseudo-Voigt band profile (50:50 Lorentzian:Gaussian) and an FWHM of 8 cm^{-1}. The Raman spectra were simulated taking into account the experimental setup (T = 207.15 K, λ = 532 nm). The band assignments were carried out by visual inspection of the normal modes with the Jmol program package.[24]

Lattice parameters and atomic coordinates of the optimized solid-state structure of Cs[Cl_3F_{10}]

Space group $P2_1/n$ (No. 14) – here, a different space group setting was used, so lattice parameters and atomic coordinates are interchanged compared to the deposited crystal structure. However, the results are of course identical.
a = 7.29891298 Å, b = 15.29673244 Å, c = 9.12132812 Å, β = 93.243220°, V = 1016.760812 Å^3

Table S7. Atomic coordinates of the optimized structure of Cs[Cl_3F_{10}].

Atom	*x*	*y*	*z*
Cs(1)	2.762404704911E−01	3.487537724337E−01	−4.851377917018E−01
Cl(1)	−2.499977788070E−01	3.955001550605E−01	3.214039278472E−01
Cl(2)	−2.064146484759E−01	1.831217546430E−01	−4.824472805740E−01
Cl(3)	−2.060477666329E−01	3.962893633638E−01	−2.590965482665E−01
F(1)	−3.040827548803E−01	3.229303714803E−01	−4.672212545441E−01
F(2)	−8.247541012499E−02	4.685669529561E−01	−3.710187149355E−01
F(3)	−2.222373885948E−01	4.480775087627E−01	1.683737183078E−01
F(4)	−4.298632094940E−01	4.701207408601E−01	3.563317591261E−01
F(5)	−6.719095268031E−02	3.257530157643E−01	2.773161315502E−01
F(6)	−3.224952817150E−01	3.292216490220E−01	−1.376579378583E−01
F(7)	−1.277514849546E−01	4.531752927419E−01	−1.171161103897E−01
F(8)	−1.259557260578E−01	8.312380724319E−02	−4.867301386579E−01
F(9)	−9.138593581981E−02	1.949154564508E−01	−3.070629551867E−01
F(10)	−3.152329059001E−01	1.650743845394E−01	3.414527937302E−01

Band assignments for the calculated Raman spectrum of $Cs[Cl_3F_{10}]$ and comparison of Raman spectra

Table S8. Band assignments for the calculated Raman spectrum (>300 cm^{-1}) of $Cs[Cl_3F_{10}]$. The notation for band assignments is the following: *ν* – stretching, *δ* – deformation, s – symmetric, as – asymmetric. The irreducible representation is given for the point group *C*2/*m* (C_{2h}) of the space group $P2_1/n$ (No. 14). The point group of the $[Cl_3F_{10}]^-$ anion in the crystal structure is C_1.

ν (calculated) / cm^{-1}	*ν* (experimental) / cm^{-1}	irrep	Assignment
787	761 & 699	A_g	ν_s – Symmetric stretching of F_{trans}–Cl
779		B_g	
758		B_g	
719		A_g	
719		B_g	
715		A_g	
650	not observed	B_g	ν_{as} – Antisymmetric stretching of F_{cis}–Cl
645		B_g	
639		A_g	
619		B_g	
613		A_g	
586		A_g	
510	498	A_g	ν_s – Symmetric stretching of F_{cis}–Cl coupled with the central μ_3-F atom
509		B_g	
509		A_g	
508		B_g	
503		A_g	
503		B_g	
381	not observed	B_g	*δ* – scissoring of F_{trans}–Cl, coupled with the central μ_3-F atom
381		A_g	
377		B_g	
376		B_g	
373		B_g	
372		A_g	
367	334	B_g	*δ* – out-of-plane bending of the ClF_3 groups, coupled with the central μ_3-F atom
364		A_g	
363		B_g	
361		A_g	
360		A_g	
360		B_g	
341		B_g	
329		A_g	
323	315	B_g	*δ* – scissoring of F_{cis}–Cl, coupled with the central μ_3-F atom
316		A_g	
315		B_g	
313		A_g	

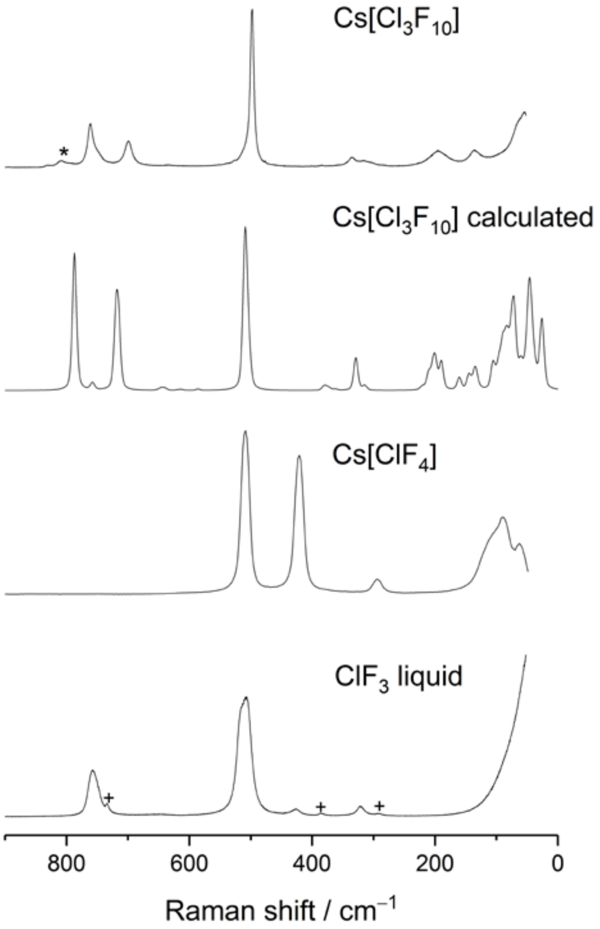

Figure S5. Comparison of experimental and calculated Raman spectra. **$Cs[Cl_3F_{10}]$:** Experimental high-resolution Raman spectrum of crystalline $Cs[Cl_3F_{10}]$ measured at ca. –66 °C under the perfluoropolyether Galden HT270. The asterisk denotes a band caused by Galden HT270. **$Cs[Cl_3F_{10}]$ calculated:** Calculated (DFT-PBE0) solid-state Raman spectrum of $Cs[Cl_3F_{10}]$ taking into account the experimental temperature of –66 °C and the used laser wavelength of 532 nm. **$Cs[ClF_4]$:** Experimental low-resolution Raman spectrum of powdered $Cs[ClF_4]$ measured in a glass vial. **ClF_3 liquid:** Experimental high-resolution Raman spectrum of liquid ClF_3 measured in an FEP (fluorinated ethylene propylene copolymer) sample tube. The plus signs denote bands that are caused by the FEP tube. The Raman intensities are given in arbitrary units.

References

[1] *APEX3*, Bruker AXS Inc., Madison, Wisconsin, USA, **2018**.
[2] G. M. Sheldrick, *CELL_NOW*, Göttingen (Germany), **2008**.
[3] G. M. Sheldrick, *Acta Crystallogr., Sect. A: Found. Adv.* **2015**, *71*, 3–8.
[4] G. M. Sheldrick, *Acta Crystallogr., Sect. C: Struct. Chem.* **2015**, *71*, 3–8.
[5] S. I. Ivlev, M. Conrad, F. Kraus, *Z. Kristallogr. - Cryst. Mater.* **2019**, *234*, 415–418.
[6] K. Brandenburg, H. Putz, *Diamond - Crystal and Molecular Structure Visualization*, Crystal Impact GbR, Bonn, **2019**.
[7] R. Ahlrichs, M. Bär, M. Häser, H. Horn, C. Kölmel, *Chem. Phys. Lett.* **1989**, *162*, 165–169.
[8] *TURBOMOLE V7.3, a Development of University of Karlsruhe and Forschungszentrum Karlsruhe GmbH, 1989-2007, TURBOMOLE GmbH, since 2007.*, **2018**.
[9] F. Weigend, R. Ahlrichs, *Phys. Chem. Chem. Phys.* **2005**, *7*, 3297–3305.
[10] K. Eichkorn, O. Treutler, H. Öhm, M. Häser, R. Ahlrichs, *Chem. Phys. Lett.* **1995**, *240*, 283–290.
[11] F. Weigend, *Phys. Chem. Chem. Phys.* **2006**, *8*, 1057–1065.
[12] G. Knizia, *J. Chem. Theory Comput.* **2013**, *9*, 4834–4843.
[13] G. Knizia, J. E. M. N. Klein, *Angew. Chem. Int. Ed.* **2015**, *54*, 5518–5522.
[14] J. P. Perdew, K. Burke, M. Ernzerhof, *Phys. Rev. Lett.* **1996**, *77*, 3865–3868.
[15] C. Adamo, V. Barone, *J. Chem. Phys.* **1999**, *110*, 6158–6170.
[16] V. Sivchik, R. K. Sarker, Z.-Y. Liu, K.-Y. Chung, E. V. Grachova, A. J. Karttunen, P.-T. Chou, I. O. Koshevoy, *Chem. Eur. J.* **2018**, *24*, 11475–11484.
[17] R. E. Stene, B. Scheibe, A. J. Karttunen, W. Petry, F. Kraus, *Eur. J. Inorg. Chem.* **2019**, *2019*, 3672–3682.
[18] A. J. Karttunen, T. Tynell, M. Karppinen, *J. Phys. Chem. C* **2015**, *119*, 13105–13114.
[19] R. Dovesi, A. Erba, R. Orlando, C. M. Zicovich-Wilson, B. Civalleri, L. Maschio, M. Rérat, S. Casassa, J. Baima, S. Salustro, et al., *Wiley Interdiscip. Rev.: Comput. Mol. Sci.* **2018**, e1360.
[20] C. M. Zicovich-Wilson, F. Pascale, C. Roetti, V. R. Saunders, R. Orlando, R. Dovesi, *J. Comput. Chem.* **2004**, *25*, 1873–1881.
[21] F. Pascale, C. M. Zicovich-Wilson, F. López Gejo, B. Civalleri, R. Orlando, R. Dovesi, *J. Comput. Chem.* **2004**, *25*, 888–897.
[22] L. Maschio, B. Kirtman, M. Rérat, R. Orlando, R. Dovesi, *J. Chem. Phys.* **2013**, *139*, 164101.
[23] L. Maschio, B. Kirtman, M. Rérat, R. Orlando, R. Dovesi, *J. Chem. Phys.* **2013**, *139*, 164102.
[24] *Jmol: An Open-Source Java Viewer for Chemical Structures in 3D. Http://Www.Jmol.Org/*, Jmol Team, **2019**.

Author Contributions

Benjamin Scheibe: Planning and conducting the experiments, main data acquisition and interpretation, quantum-chemical solid-state calculations, manuscript preparation.

Antti J. Karttunen: Quantum-chemical gas-phase calculations and solid-state calculations in CsF polymorphs, Quantum-chemical gas-phase calculations, CRYSTAL17 basis set development, intrinsic atomic orbital (IAO) and intrinsic bond orbital (IBO) analysis, manuscript preparation.

Ulrich Müller: Data interpretation, preparation of the Bärnighausen tree, crystallographic considerations, manuscript preparation.

Florian Kraus: Project administration, data interpretation, structure chemistry, manuscript preparation.
